# How artificial intelligence can help us ‘Choose Wisely’

**DOI:** 10.1186/s42234-021-00066-8

**Published:** 2021-04-21

**Authors:** Nishila Mehta, Karen Born, Benjamin Fine

**Affiliations:** 1 Temerty Faculty of Medicine, King’s College Cir, Toronto, ON M5S 1A8 Canada; 2 Unity Health Toronto, 30 Bond Street, Toronto, Ontario M5B 1W8 Canada; 3 Institute for Health Policy, Management and Evaluation, University of Toronto, 155 College St 4th Floor, Toronto, ON M5T 3M6 Canada; 4 Department of Diagnostic Imaging and Institute for Better Health, Trillium Health Partners, 2200 Eglinton Ave W, Mississauga, ON L5M 2N1 Canada; 5 WCH Institute for Health System Solutions and Virtual Care (WIHV), Women’s College Hospital, 76 Grenville St, Toronto, ON M5S 1B2 Canada

**Keywords:** Clinical decision support, Artificial intelligence, Resource stewardship, Quality improvement

## Abstract

The overuse of low value medical tests and treatments drives costs and patient harm. Efforts to address overuse, such as Choosing Wisely campaigns, typically rely on passive implementation strategies- a form of low reliability system change. Embedding guidelines into clinical decision support (CDS) software is a higher leverage approach to provide ordering suggestions through an interface embedded within the clinical workflow. Growth in computing power is increasingly enabling artificial intelligence (AI) to augment such decision making tools. This article offers a roadmap of opportunities for AI-enabled CDS to reduce overuse, which are presented according to a patient’s journey of care.

## Background

Overuse of tests, treatments and procedures is a complex quality challenge for health care systems, with estimates suggesting that 30% of all health care delivered in Canada and the United States offers no clinical value to patients and can potentially lead to harm (IOM (Institute of Medicine), 2013; Canadian Institute for Health Information, 2017; Braithwaite et al., 2020; Pathirana et al., 2017). Overuse - where expected health benefits of care do not clearly exceed negative consequences (risk of harm, pain, misleading results etc.) - is driven by the interplay of several complex factors including clinician practice patterns, new diagnostic technologies, patient expectations, and funding incentives (Pathirana et al., 2017).

Research suggests that efforts to reduce overuse will require a combination of system-level strategies alongside bottom-up efforts to target increased awareness of overuse and how the clinician-patient interaction can drive this quality problem (Mafi & Parchman, 2018). Choosing Wisely campaigns in countries around the world have raised awareness about overuse by partnering with national clinician societies to develop specialty-specific recommendations around tests, treatments and procedures which are commonly overused (Choosing Wisely, 2019a). Clinicians and patients are then tasked with implementing these recommendations (a form of passive education) into day-to-day clinical practice, which is a recognized form of low reliability system change (Institution for Safe Medication Practices, 2020). Clinicians find it difficult to implement campaign recommendations given the demands of the practice environment and systems which incentivize overuse and importantly, the uniqueness of each patient which rarely perfectly match clinical vignettes portrayed in guidelines (Grimshaw et al., 2020; Embrett & Randall, 2018; Gupta et al., 2017).

Consider the common scenario of a general practice physician seeing a patient presenting with a headache in a primary care setting. First, the relevant clinical history and physical exam information must be collected. Then, to reach a management decision, such as whether or not to order imaging, the physician must reconcile their clinical experience (“most patients like this that I have seen turn out to be fine, but I once saw one with a brain tumor present as a headache”) with recall of evidence-based recommendations (“what are those criteria for imaging again?”) to arrive at a clinical decision. Consider if instead, the reassurance of an expert in headache - one who has seen thousands of patients with similar clinical presentations - could be applied to inform decision-making for this patient. Increasingly, this is becoming possible. The digitization of medical records, exponential growth in computing power and availability of ever-advancing machine learning algorithms over the past decade are making deploying an AI-enabled “expert” tool to assist each physician-patient interaction increasingly possible (Office of the National Coordinator for Health Information Technology, 2019; Topol, 2019; Jiang et al., 2017).

In this article, we begin by exploring how data can serve as a new foundation for clinical decision support (CDS) tools. We then outline opportunities for AI-enabled clinical decision making tools to augment health system efforts to promote high value care. This roadmap of problems and applications can help guide the policy, clinical, software development, and data science communities to address via AI-enabled technologies and supporting to reduce overuse and drive value.

### Advances and opportunities in clinical decision support

There is a well-documented and wide chasm between research and clinical practice; it can take decades for evidence-based practices which are detailed in guidelines and grounded in randomized controlled trials research to reach the bedside (Bero et al., 1998).

Embedding guidelines into CDS software is a higher leverage approach to encourage evidence-based decision making through an interface embedded within the clinical workflow. These tools are designed to be used interactively in reaching clinical decisions, and have been widely incorporated in healthcare for various applications including preventing adverse events or medical errors (e.g. drug interactions) and reducing healthcare costs (Middleton et al., 2016). The underlying analytic methodologies employed by these technologies have evolved over time, from early “rule-based” systems to more sophisticated methods today employing statistical machine learning (Montani & Striani, 2019). Newer methods are enabling CDS to move beyond *knowledge-based* approaches (e.g. applying relevant guidelines to a patient), to *data-driven* approaches, which take advantage of the large volumes of patient data being stored in electronic formats to identify patterns in a “bottom up” fashion and make patient-specific recommendations (Montani & Striani, 2019; Sutton et al., 2020). It is becoming possible to predict which patients can benefit from interventions, and which will not, through personalized patient data drawn from sources such as electronic medical records (Yu et al., 2018). Enabling precise and patient specific recommendations will also aid in addressing alert fatigue, a challenge which has plagued the implementation of CDS software (Sutton et al., 2020).

**Fig. 1:**
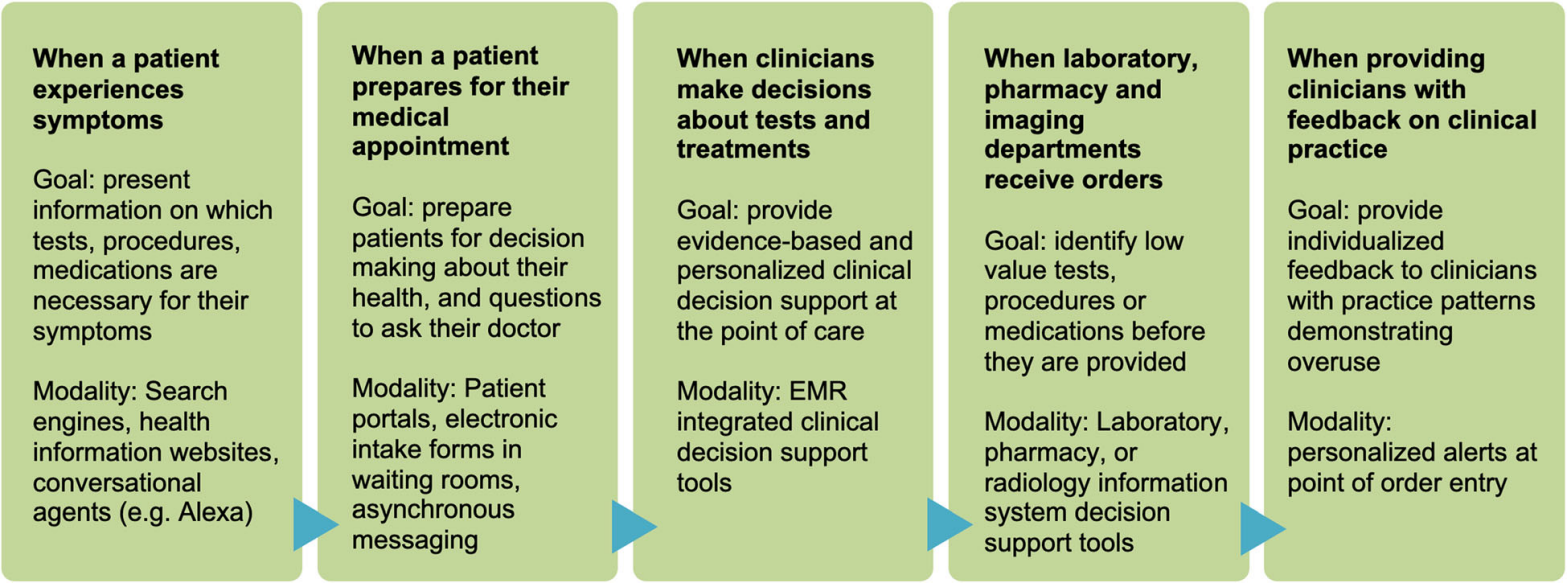
How AI-enabled CDS tools can reduce overuse at various stages of a patient’s care journey

This approach is being applied in the growing field of precision medicine which uses unique patient features to identify, for example, which patients should be prescribed a drug based on their clinical or genetic features (Mesko, 2017). Analogously, AI could help clinicians take a more precise approach to reducing overuse (Shortliffe & Sepúlveda, 2018). As a hint of this potential, a recent study trained a machine learning model on past patients’ clinical data from their EMR coupled with annotated computed tomography (CT) results to derive patient-specific risk scores for pulmonary embolism that reduced the need for CT in new patients by 60% (Banerjee et al., 2019). In the next section, we explore how modern data and computing can be applied to reduce overuse during common scenarios along a care journey (Fig. 1).

## Opportunities for AI-enabled CDS

### Stage 1: when a patient experiences symptoms

When patients experience a symptom, it has become common to seek out the cause of such symptoms through advanced testing, which is often costly but may also be of low clinical value (Mira et al., 2018). This is bolstered through access to health information on the internet, where often lists of diagnoses are found alongside tests to rule them out. Advances in natural language processing (NLP), a branch of artificial intelligence, present an opportunity to tackle overuse at this stage. Evidence has shown that patient-facing interventions which incorporate health education messaging and recommend alternative behaviours are effective in reducing overuse (Lin et al., 2020). Sources where patients frequently seek information, such as search engines or health information websites, could employ simple NLP solutions to present resource aware recommendations when patients search for symptoms. Google, for example, presented treatment options to patients related to COVID-19 searches in 2020; search engine providers could perform a similar public service by helping patients avoid low value choices (Google, 2020). A variation of this could take advantage of AI-enabled conversational agents or ‘chatbots’ (computer programs trained to mimic human conversation), which are increasingly being used to deliver evidence-based information to patients when they need it, to begin early discussions of which tests and treatments are needed for their symptoms (Laranjo et al., 2018). For example, a patient in the future could ask “will I need an x-ray for my back pain?”; a response based on a relevant Choosing Wisely recommendation could reassure that “99% of patients like you find no benefit from imaging at this point”, and describe harms from unnecessary imaging (Hall et al., 2021). As an early sign of what could become more commonplace, during the COVID-19 pandemic certain leading health systems leveraged AI-powered mobile tools to screen and triage patients at large volumes and low cost as a way to divert care away from overwhelmed emergency departments (Harvard Business Review, 2021).

### Stage 2: when a patient prepares for a medical appointment

Choosing Wisely campaigns create patient-facing materials, which explain commonly overused interventions and encourage patients to ask their doctors questions about the necessity of tests and treatments (Born et al., 2017; Choosing Wisely, n.d.). Dissemination of evidence-based health information to patients is challenging due to the vast quantity of information available and limited clinician time to connect patients to relevant resources. A growing opportunity to harness AI to enhance patient education around low value care is through patient portals, secure websites that provide patients access to their health information from anywhere, which are being widely adopted by healthcare organizations (Ammenwerth et al., 2012). For example, an AI-based recommender system integrated into a patient portal could mine imaging appropriateness criteria to help patients decide if they have clinical features that might warrant imaging (e.g. red flags in low back pain), and present factors for patients to consider in their decision, such as cost, radiation dose, time off work, etc. (Sahoo et al., 2019) Another opportunity is waiting rooms of ambulatory clinics, where providing patients with educational materials on common overuse topics improves patient knowledge around unnecessary care (Silverstein et al., 2016). The increasing use of tablets and digital kiosks for intake forms in waiting rooms can be combined with a simple NLP-based solution to recognize text associated with frequently overused tests, procedures and medications on patient intake forms, and present relevant questions and considerations for patients. For example, patients presenting with a chief complaint of sinusitis might be immediately presented with patient-facing information about appropriate indications for antibiotics for upper respiratory tract infections which they might read prior to their appointment (Silverstein et al., 2016).

### Stage 3: when clinicians and patients make decisions about Tests & Treatments

Real time entry of patient data into EMRs during clinical encounters has become commonplace. The field of CDS has leveraged this to present evidence-based recommendations to clinicians at the point of care. How can CDS systems be leveraged to prevent overuse, and how can AI help? A vast number of recommendations for clinicians exist to help reduce unnecessary tests and treatments- Choosing Wisely campaigns in more than 25 countries have developed thousands of recommendations (Choosing Wisely, 2019b). Presently, most CDS tools used in practice are rule-based and not patient specific: if a physician orders a lumbar spine MRI but has not checked the box for “trauma”, “malignancy” or “radiculopathy”, the CDS will suggest the test not be ordered. As a result of this and other challenges like alert fatigue, only small to modest changes in physician behavior are typically observed upon CDS system implementation (Shojania et al., 2009; Kwan et al., 2020). As CDS systems utilize AI based methods to enable personalized predictions with improved accuracy, they can more precisely match recommendations to patient contexts (Topol, 2019; Jiang et al., 2017). AI algorithms, such as the Pulmonary Embolism Result Forecast Model, can be deployed within CDS software enabling the use of case-specific recommendations; if, in the case of a patient presenting to the emergency department with intermediate pretest probability of pulmonary embolism, the patient-specific likelihood of a positive CT Pulmonary Angiogram study is now known, the patient and provider may engage in decision-making that could reduce low value imaging in a sizable portion of patients (Banerjee et al., 2019). More accurately matching recommendations to patient contexts can help reduce the overall number of alerts for clinicians and mitigate alert fatigue (Chen et al., 2020; Khreis et al., 2019).

### Stage 4: when laboratory, pharmacy and imaging departments receive orders

In many health systems, testing and treatment is generated through orders submitted to laboratory, pharmacy or radiology information systems. CDS is being applied to augment decision making at the of order entry in these information systems (Berner, 2009). Current systems are not context-aware; they simply present pre-defined guidelines based on the ordered examination (e.g. MRI brain) and are not aware of the patient’s clinical context from the EMR. As a result, these systems offer general population-level, not patient-specific, recommendations. If CDS software was aware of clinical EMR data in real time, these systems could use patients’ data, such as their specific clinical condition and comorbidities, to more accurately identify orders that may not fall in line with Choosing Wisely or other appropriateness guidelines when received by the laboratory, pharmacy, imaging departments. Once flagged, these orders might be rejected where there is strong evidence of inappropriateness (e.g. routine daily blood tests for clinically stable hospitalized inpatients), or be flagged for expert clinician review as a second opinion (e.g. blood transfusion on a relatively stable inpatient).

### Stage 5: when providing clinicians with feedback on clinical practice

Currently, most clinicians receive feedback from individual patient encounters, or on aggregate (e.g. mammography recall rate). Largely untapped is the EMR data that captures every clinical decision made by a physician for each patient. Physicians’ ordering patterns for clusters of patients with similar characteristics can be collected and analyzed. This data can be used to provide feedback on how clinicians ordering behavior compares to their peers, a strategy which has been shown to be highly effective in promoting appropriate resource use (Zafar et al., 2019). For example, it would be relatively easy to use audit and feedback strategies using data of MRI ordering for patients with uncomplicated headache (clustered based on EMR data) relative to their peers. The emerging idea of using AI to personalize choice architecture in the field of behavioural economics could be tailored to physicians by integrating specific physicians’ habits and trends to create personalized digital nudges towards adhering to recommendations at the point of clinical decision making or computerized order entry (Thaler & Sunstein, 2008; Choosing Wisely, 2019c; Hrnjic & Tomczak, 2019; Karlsen & Andersen, 2019). From a health systems perspective, these data can be combined with system level data to assess performance on common measures of overuse, and incentivize resource stewardship for example through public reporting of hospitals’ resource use appropriateness and tying performance measures to funding or reimbursemnt (Doll & Patel, 2015).

## Challenges and limitations

The future of medicine offers many new possibilities for computer intelligence to scale expertise to make it easier for clinicians and patients to make choices that drive high value care.

However, confronting new possibilities for reducing overuse through AI will come with considerable challenges currently facing all augmented medical decision making (Celi et al., 2019; Maddox et al., 2019). First and foremost, relevant data from EMRs must be available to mine in order to develop predictions. This means health systems need to integrate their multiple health information systems and EMR vendors need to open up their data for sharing. While there is progress on this front – for example, the development of health information communication standards such as FHIR - EMR data remains inaccessible in many commercial applications and siloed in many institutions. Building machine learning algorithms using health data also involves grappling with issues of data quality, including data accuracy and missingness, and identifying and mitigating bias in predictions. Deploying an algorithm then becomes a software challenge: on the back end, a predictive model must integrate with each EMR, which is a costly custom development and business agreement challenge. On the front end, we must ensure the recommendation is delivered in a way that will promote better decision making and does not disrupt clinical workflows in a way that increases costs or delays patient care; a poorly designed user interface by itself can drive overuse (Vaughn & Linder, 2018; Emanuel & Wachter, 2019). These systems then need to be monitored and adjusted in production, completing the AI product lifecycle and ensuring quality and safety- an entirely new practice in healthcare (Geis et al., 2019).

Overuse is also driven in part by clinician practice patterns and habits. In many jurisdictions, due to the fear of litigation clinicians will order more diagnostic tests for example in the face of uncertainty. Physician (and patients’) comfort with the levels of uncertainty in algorithms’ predictions, and liability issues with this technology when errors occur, are a new legal and ethical consideration. How much physicians and patients trust the output and recommendations of algorithms will also be a determining factor in their ability to modify clinical practice to prevent overuse (Asan et al., 2020; Nundy et al., 2019).

Overall, physician ordering behavior, habits and practice can be difficult to change and require a number of multi-faceted strategies including effective leadership and change management, audit and feedback, clinician education, standards and policies, user-centered software design, thoughtful nudge design and automation as appropriate (Grimshaw et al., 2020; Gupta et al., 2017;Vaughn & Linder, 2018). Simply offering access to relevant information may not be sufficient to change physician habits and behaviors.

## Conclusion

Despite several challenges and unknowns, the sustainability implications of the overuse problem to health systems necessitate solutions. The computer science and medical communities can combine efforts and work with policy makers and software vendors to build and deploy AI-enabled CDS tools. Such tools have the potential to unlock opportunities to support solutions to foster high value care.

## Data Availability

Not applicable.
